# Population pharmacokinetic analysis of tobramycin in serum and ELF using data from patients with pneumonia

**DOI:** 10.1128/aac.00908-24

**Published:** 2025-04-14

**Authors:** Scott A. Van Wart, Michael Trang, M. Courtney Safir, Andrew R. Santulli, Christopher M. Rubino, Sujata M. Bhavnani

**Affiliations:** 1 Institute for Clinical Pharmacodynamics Inc 537914, Schenectady, New York, USA; 2 Enhanced Pharmacodynamics, LLC, Buffalo, New York, USA; Providence Portland Medical Center, Portland, Oregon, USA

**Keywords:** tobramycin, epithelial lining fluid, population pharmacokinetic analysis

## Abstract

Population pharmacokinetic analyses were undertaken to characterize epithelial lining fluid (ELF) penetration for tobramycin in pneumonia patients. A two-compartment model with zero-order intravenous input and first-order elimination linked to an effect site ELF compartment was utilized to determine the steady-state ELF penetration ratio of 0.51 for tobramycin. These results are useful to account for effect site exposure when performing analyses to support recommendations for aminoglycoside dosing regimens and interpretive criteria for *in vitro* susceptibility testing.

## INTRODUCTION

Drug exposure at the infection site is important when evaluating dose and/or interpretive criteria for *in vitro* susceptibility testing of antimicrobial agents against pathogens of interest. Bronchoscopy with bronchoalveolar lavage (BAL) is used to determine concentrations in the epithelial lining fluid (ELF) of the respiratory tract, which is a relevant effect site for extracellular respiratory pathogens causing acute bacterial pneumonia and infective exacerbation of chronic bronchitis (1). While aminoglycosides are known for poor lung penetration and varied lung tissue concentrations related to the anatomical site sampled (2, 3), a better understanding of the ELF penetration of aminoglycosides is useful to facilitate the population pharmacokinetic (PK) modeling and simulation that can be carried out to support the assessment of dosing regimens and susceptibility test interpretive criteria for these agents. Thus, the literature was searched for studies in which patients administered an aminoglycoside underwent serum PK sampling, and had BAL fluid samples collected at various times across the dosing interval. Priority was given to studies that provided individual dosing, PK, demographic, and renal function data.

Several studies were identified but ultimately not considered due to the collection of serum and/or BAL fluid PK samples at a single time point in all subjects rather than staggering the PK sample collection times (4–7). Such studies are limited in their utility as a result of system hysteresis, which is characterized by ELF concentration-time profiles that are delayed relative to serum concentration-time profiles. The implication of system hysteresis is that true penetration of a drug into the ELF is difficult to quantify unless sufficient PK data are available to inform ELF and serum concentration-time profiles (1).

Three studies that did fit the search criteria evaluated gentamicin, netilmicin, or tobramycin (8–10). Of these three studies which evaluated the ELF penetration for aminoglycosides in the patient population of interest (i.e., with pneumonia), the study conducted for tobramycin by Carcas et al. (10) was ultimately selected for further analysis as it provided individual demographics, renal function estimates, and most importantly, time-matched ELF and serum concentration-time data by patient across the 8-h dosing interval.

In the study by Carcas et al. (10), steady-state ELF and serum PK samples were collected from 16 adult patients with pneumonia. Tobramycin was assumed to be administered at 1 mg/kg as a 30-minute intravenous (IV) infusion every 8 h (q8h) to all patients. However, the authors stated dose adjustment was performed as necessary to obtain peak and trough concentrations of approximately 8 and <2 mg/L, respectively. Bronchoscopy with BAL was performed at least 2 days after dose adjustment to allow for steady-state serum concentrations to be reached. A BAL fluid sample was collected once for each patient at either 0.5, 2.0, 4.0, or 8.0 h after the previous tobramycin dose (four patients per time point), and urea-corrected ELF concentrations were determined. Tobramycin concentrations in serum were measured for all patients at each of the four time points.

**Fig 1:**
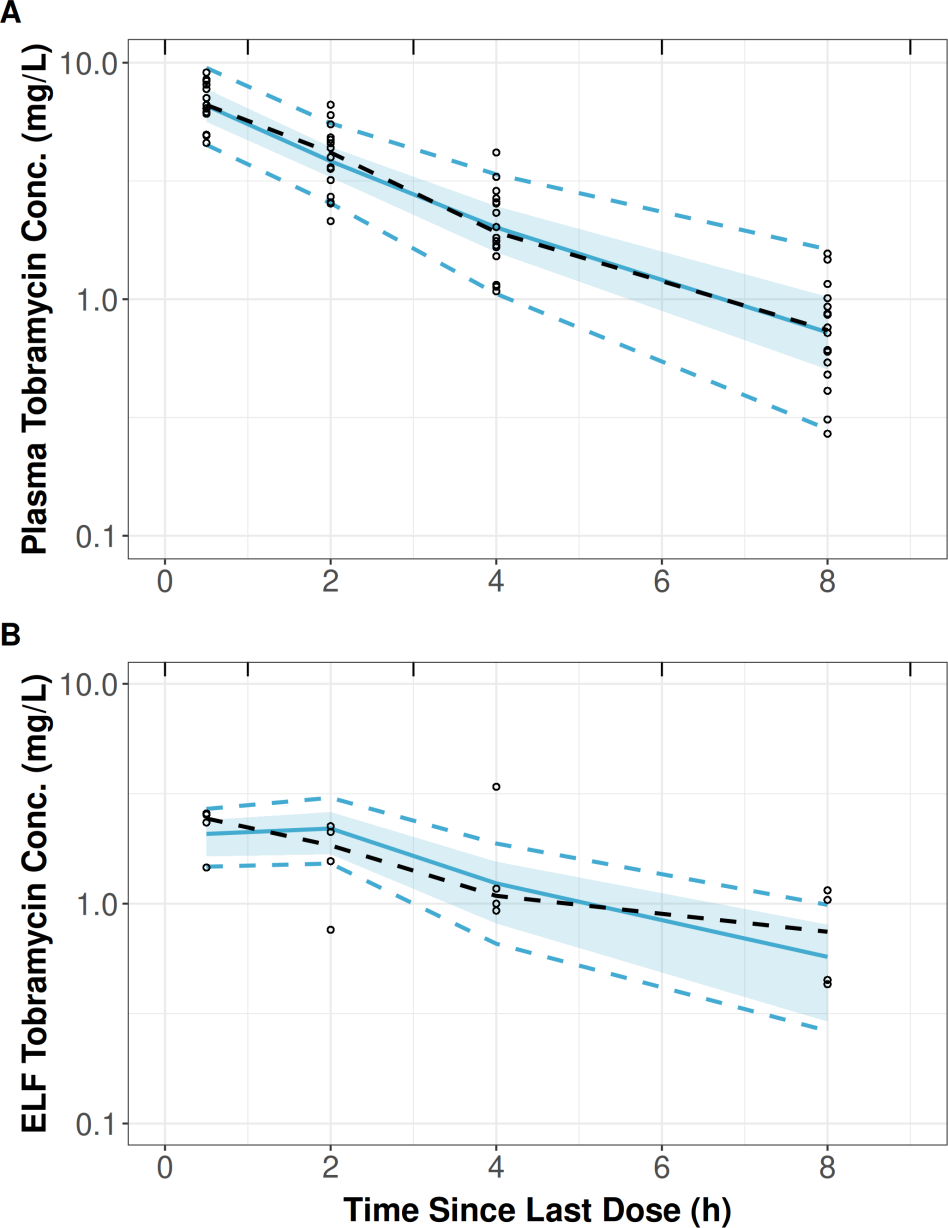
Visual predictive check for the population PK model to the tobramycin steady-state total-drug serum (A) and ELF (B) concentration-time data from pneumonia patients administered 1 mg/kg of tobramycin IV infused over 30 minutes q8h published by Carcas et al. (10). The black dashed line is the median of observed data (black dots). The solid blue line and shaded area are the overall median and 80% bootstrap confidence interval of medians for 1,000 simulations. The dashed blue lines denote the 90% prediction interval of the simulations. Bin boundaries are indicated along the upper edge of the plot panel (black line segments). ELF, epithelial lining fluid; q8h, every 8 h.

The population PK analysis was conducted using NONMEM software version 7.4 (ICON Development Solutions, Ellicott City, MD), implementing the first-order conditional estimation method with interaction (11). A two-compartment model with zero-order IV input and first-order elimination, with a linked effect site compartment for ELF, was used to simultaneously characterize the time course of tobramycin in serum and ELF upon repeated IV dosing. This structural PK model used serum drug concentrations to drive appearance in and subsequent removal of drug from the ELF without impacting the serum PK data fitting. Calculated creatinine clearance was included as a covariate on clearance using a power function. Parameter estimates for the final population PK model are shown in Table 1. The hysteresis for tobramycin concentrations in the ELF as noted in other studies (8, 9) was adequately captured, and the equilibrium half-life was calculated to be approximately 12 minutes based on the first-order elimination rate constant from the ELF compartment (*k*_30_). A visual predictive check (12) demonstrated excellent agreement between the simulated and observed data following a steady-state dose (Fig. 1), indicating that the model reasonably captured the central tendency and variability in the plasma and ELF concentration-time data and should provide reasonable predictions when performing stochastic simulations. A structural PK model diagram, goodness-of-fit plots, individual predicted overlays, and a NONMEM control stream containing the model differential equations as well as the actual analysis data set are all provided in the Supplemental Material.

**TABLE 1:** Final population PK parameter estimates based on serum and ELF PK data from patients with pneumonia published by Carcas et al. (10)^*a*^

Parameter	Final estimate	% RSE
CL (L/h) for a typical 90 mL/min/1.73 m^2^ patient CL-CLcr exponent	3.26 0.685	6.41 31.0
Vc (L)^*b*^	10.4	7.03
CLd (L/h)	0.518	25.8
Vp (L)^*b*^	10.4	7.03
*k*_13_ (h^−1^)	1.81	23.1
*k*_30_ (h^−1^)	3.69	38.9
*ω* ^2^_CL_^*c*^	0.0488 (22.4% CV)	31.6
*ω* ^2^_Vc_^*c*^	0.0488 (22.4% CV)	31.6
*ω* ^2^_*k*30_^*c*^	0.0488 (22.4% CV)	31.6
*σ*^2^ CCV for serum data	0.0299 (17.3% CV)	36.3
*σ*^2^ Additive for ELF data	0.264 (0.514 SD)	61.6

^*a*^ CCV, constant coefficient of variation; CL, clearance; CLcr, calculated creatinine clearance; CLd, distributional clearance; CV, coefficient of variation; ELF, epithelial lining fluid; *k*_13_, first-order distribution rate constant from the central compartment to the ELF compartment; *k*_30_, first-order elimination rate constant from the ELF compartment; *ω*^2^, interindividual variability; *σ*^2^, residual variability; q8h; every 8 h; RSE, relative standard error; SD, standard deviation; Vc, volume of distribution in the central compartment; Vp, volume of distribution in the peripheral compartment.

^*b*^ Due to model parameter identifiability concerns, Vc and Vp were constrained to have the same value during model fitting. The resulting volume of distribution at a steady-state of 20.8 L (or 0.297 L/kg for a 70-kg patient) was within the range of values (0.28–0.45 L/kg) previously reported (13–18).

^*c*^ For model reduction purposes, the magnitude of the interindividual variability for CL, Vc, and *k*_30_ was constrained to have the same estimated value during model fitting.

Using the final population PK model, a simulation of 1,000 patients with pneumonia receiving tobramycin as a 1 mg/kg IV infusion for 30 minutes q8h for 14 days was performed after bootstrapping from the population of model development patients. Total-drug area under the concentration-time curve from time 0 to 8 h (AUC_0–8_) was calculated via numerical integration and maximum drug concentration (*C*_max_) by direct observation using intensive serum and ELF concentration-time data. The ELF:serum penetration ratio was computed by dividing the ELF AUC_0–8_ by the serum AUC_0–8_. Free-drug serum AUC_0–8_ was not evaluated since the protein binding for tobramycin was reported to be negligible (19). Summary statistics of tobramycin steady-state total-drug serum and ELF *C*_max_, time to *C*_max_ (*T*_max_), and AUC_0–8_ values, as well as the ELF penetration ratio, are presented in Table 2 . The mean ± standard deviation (SD) of the simulated *T*_max_ in ELF was 1.0 ± 0.1 h. The mean ± SD total-drug ELF penetration ratio for tobramycin was determined to be 0.51 ± 0.12. This mean AUC-based ELF penetration ratio of 0.51 was considered to be more informative than the time-matched concentration ratio reported to be 0.30 at 0.5 h, 0.42 at 2.0 h, 0.64 at 4.0 h, and 1.53 at 8.0 h by Carcas et al. (10).

**TABLE 2:** Summary statistics of tobramycin steady-state total-drug serum and ELF *C*_max_, AUC_0–8_, and ELF AUC_0–8_ penetration ratios simulated using data from 1,000 virtual pneumonia patients administered 1 mg/kg of tobramycin IV infused over 30 minutes q8h^*a*^

Metric	Mean	SD	Median	Minimum­, maximum
Serum *C*_max_ (mg/L)	6.60	0.76	6.56	4.78, 9.64
ELF *C*_max_ (mg/L)	2.79	0.65	2.70	1.31, 6.08
ELF *T*_max_ (h)	1.0	0.1	1.0	0.7, 1.4
Serum AUC_0–8_ (mg∙h/L)	20.5	5.2	20.0	8.44, 45.1
ELF AUC_0–8_ (mg∙h/L)	10.3	3.5	9.75	3.92, 30.1
Ratio of ELF AUC_0–8_:serum AUC_0–8_	0.51	0.12	0.49	0.23, 1.02

^*a*^ AUC_0–8_, area under the concentration-time curve from time 0 to 8 h; *C*_max_, maximum serum concentration; ELF, epithelial lining fluid; q8h, every 8 h; SD, standard deviation; *T*_max_, time of maximum serum concentration.

Although published data based on non-comparative studies assessing aminoglycoside ELF penetration (5, 8, 9) and other more recent analyses (13) have demonstrated that ELF penetration is highly variable (50%–100%), our analysis based on informative individual data collected for tobramycin suggested the ELF penetration for aminoglycosides might conservatively be closer to the lower end of that range. Future analyses of such data across aminoglycosides, including newer agents that undergo clinical development and require this information for selecting effective dosing regimens, will be useful to confirm this assumption. Such information regarding ELF penetration ratio is useful for older antimicrobial agents like the aminoglycosides, which have undergone reassessment of dosing regimens and interpretive criteria for *in vitro* susceptibility testing (20, 21).

## SUPPLEMENTAL MATERIAL

Supplemental material aac.00908-24-s0001.pdf Structural PK model diagram, goodness-of-fit plots, individual predicted overlays, and a NONMEM control stream containing the model differential equations as well as the actual analysis data set.

ASM does not own the copyrights to Supplemental Material that may be linked to, or accessed through, an article. The authors have granted ASM a non-exclusive, world-wide license to publish the Supplemental Material files. Please contact the corresponding author directly for reuse.
