# Supplementary material for: Population pharmacokinetic analysis of tobramycin in serum and ELF using data from patients with pneumonia
Source: Antimicrob Agents Chemother. 2025 Apr 14;69(5):e00908-24. doi: 10.1128/aac.00908-24 (PMC12057349; doi:10.1128/aac.00908-24)
Supplement: Supplemental material — Structural PK model diagram, goodness-of-fit plots, individual predicted overlays, and a NONMEM control stream containing the model differential equations as well as the actual analysis data set. [file aac.00908-24-s0001.pdf]

Figure S1. Diagram of population PK model used to simultaneously characterize serum and ELF tobramycin concentrations

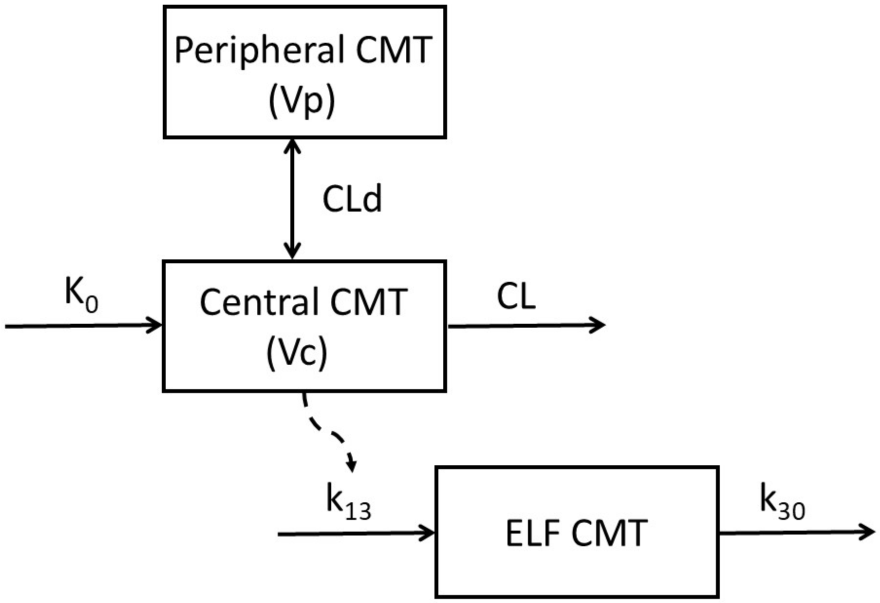

CL, clearance; CLd, distributional clearance; CMT, compartment; ELF, epithelial lining fluid; K_0, zero-order intravenous infusion rate constant; k_{13}, first-order distribution rate constant from the central compartment to the ELF compartment; k_{30}, first-order elimination rate constant from the ELF compartment; Vc, volume of distribution in the central compartment; Vp, volume of distribution in the peripheral compartment.

Figure S2. Goodness-of-fit plots for serum tobramycin concentration-time data

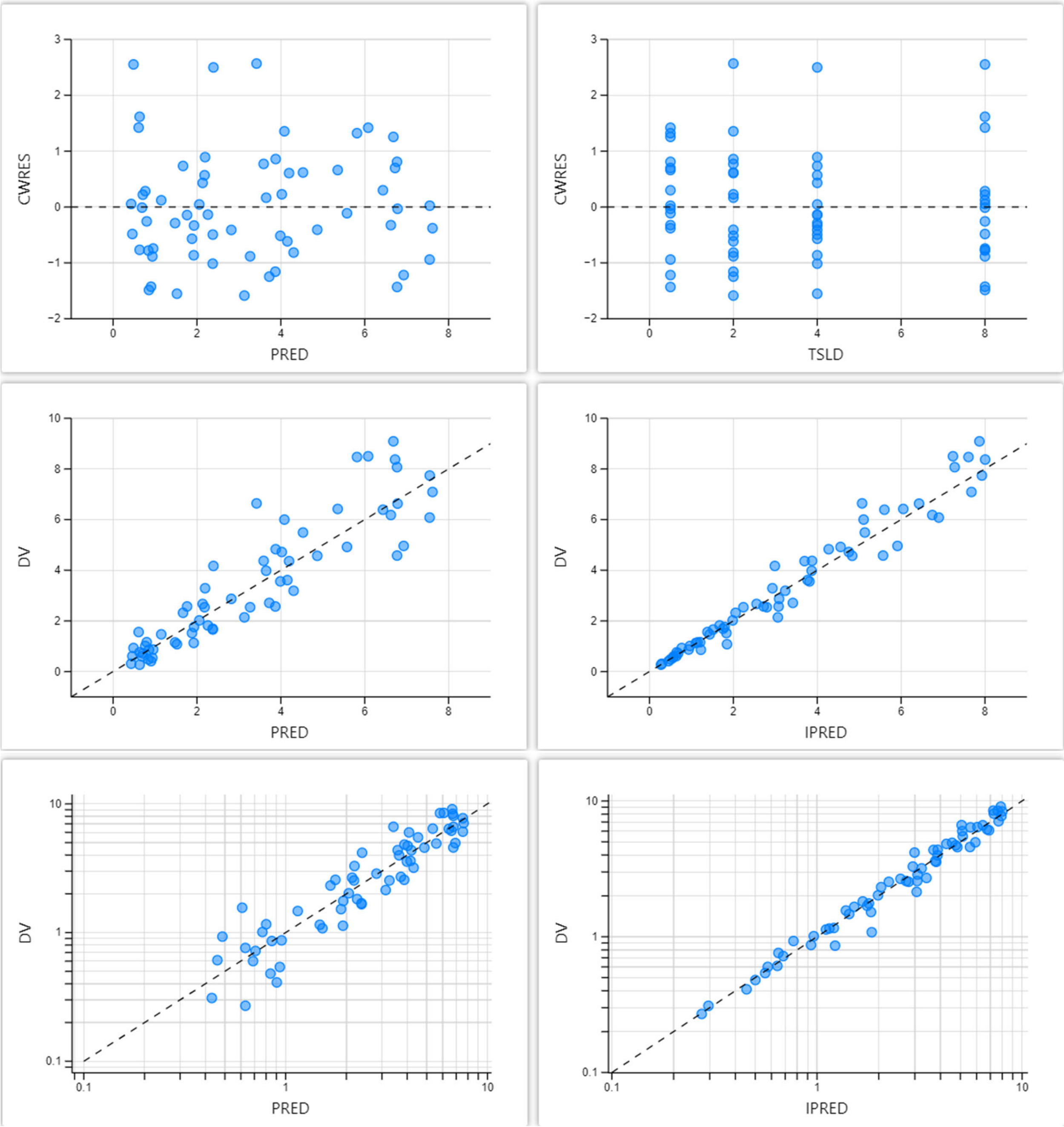

CWRES, conditional weighted residuals; DV, observed serum drug concentration; IPRED, individual predicted serum drug concentration; PRED, predicted serum drug concentration; TSLD, time since last dose.

Figure S3. Goodness-of-fit plots for ELF tobramycin concentration-time data

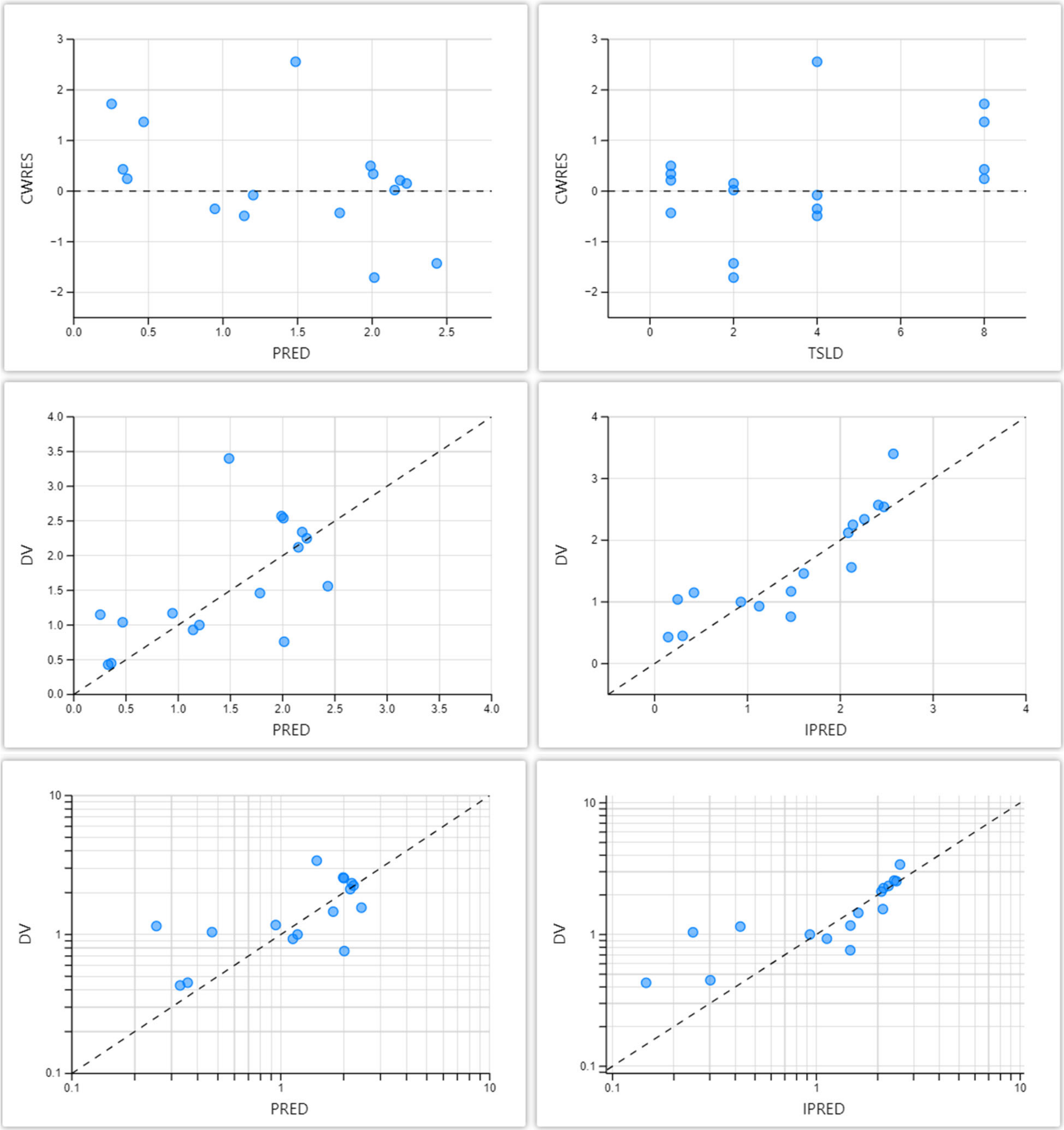

CWRES, conditional weighted residuals; DV, observed ELF drug concentration; ELF, epithelial lining fluid; IPRED, individual predicted ELF drug concentration; PRED, predicted ELF drug concentration; TSLD, time since last dose.

Figure S4. Individual fit plots for serum tobramycin concentration-time data

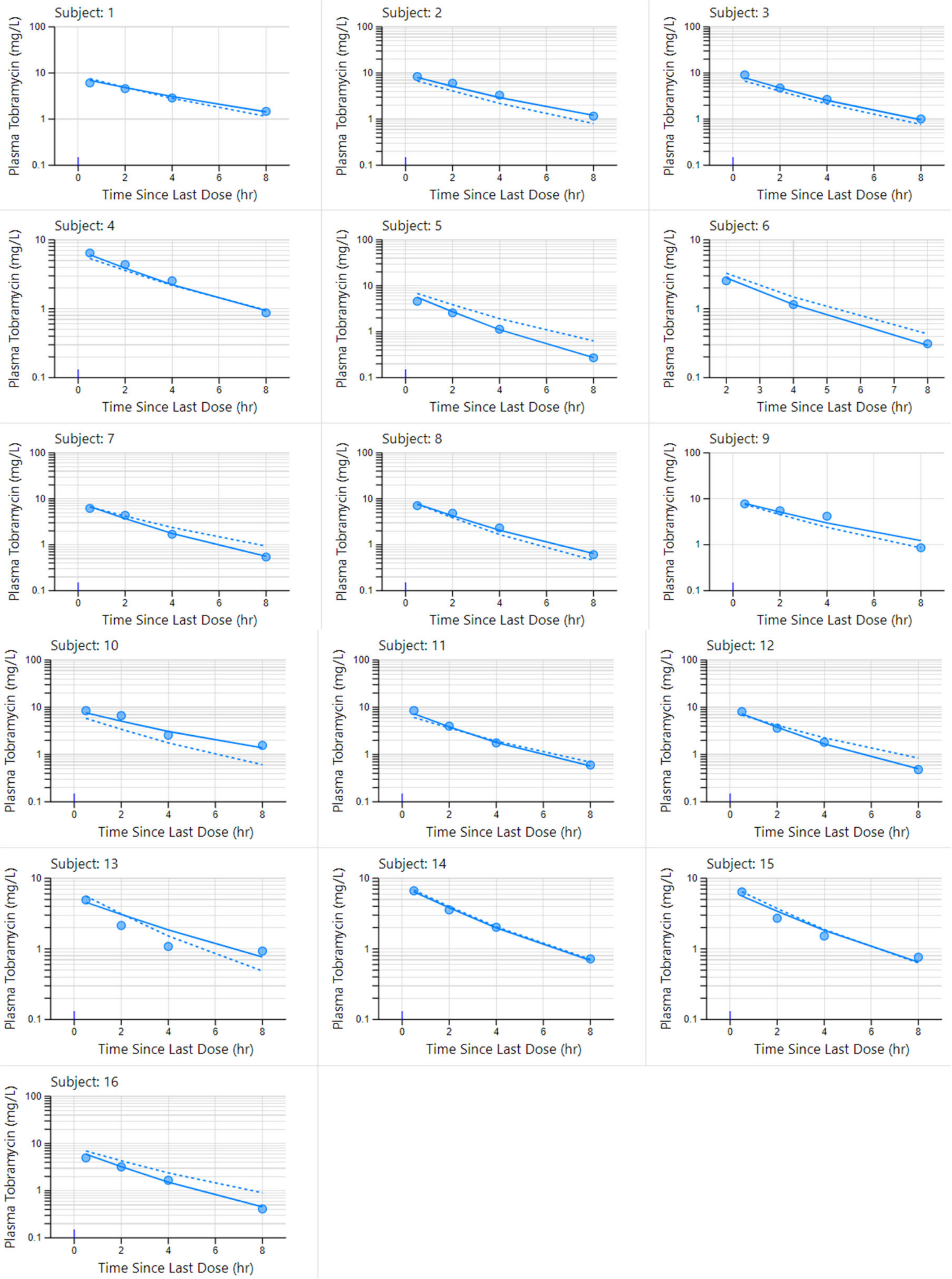

Dots=Observed data, solid line = individual predictions; dashed line = population predictions.
hr, hours; L, liters; mg, milligrams.

NONMEM control stream for the population PK model for tobramycin

```
$PROB Population PK Model for tobramycin in plasma and ELF

$ABBREVIATED DERIV2=NO

$INPUT ID TIME TSLD AMT RATE ADDL II DV CMT EVID MDV WTKG AGE SEXF HTCM SCR

$DATA ../../data/tobramycin-elf-data-31oct2024.csv
  IGNORE=I

$SUBROUTINES ADVAN13 TRANS1 TOL=6 SUBROUTINES=D

$MODEL
  COMP=(PLASMA,DEFDOSE,DEFOBS)
  COMP=(PERIPH,NODOSE)
  COMP=(ELF,NODOSE)

$PK

  R1 = RATE

  BSA=0.0235*(WTKG**0.51456)*(HTCM**0.42246)     ; Gehan and George
  IF(SEXF.EQ.0) THEN
   CLCR = (140.0-AGE)*WTKG/(72.0*SCR)*(1.73/BSA) ; Males, in mL/min/1.73 m^2
  ELSE
   CLCR = ((140.0-AGE)*WTKG/(72.0*SCR))*0.85     ; Females, mL/min/1.73 m^2
  ENDIF

  MU_1 = LOG(THETA(1))+THETA(6)*LOG(CLCR/90.0)
  CL = EXP(MU_1+ETA(1))

  TVVC = THETA(2)
  MU_2 = LOG(TVVC)
  VC = EXP(MU_2+ETA(2))

  CLD = THETA(3)

  VP = TVVC
  K10 = CL/VC
  K12 = CLD/VC
  K21 = CLD/VP
  K13 = THETA(4)

  TVK30 = THETA(5)
  MU_3 = LOG(TVK30)
  K30 = EXP(MU_3+ETA(3))

  S1 = VC ; dose=mg, conc=mg/L
  S2 = VP
  S3 = VC

$DES

  DADT(1) = K21*A(2) - K12*A(1) - K10*A(1)
  DADT(2) = K12*A(1) - K21*A(2)
  DADT(3) = K13*A(1) - K30*A(3)

$ERROR

  CP = A(1)/S1
  CPERIPH = A(2)/S2
  CELF = A(3)/S3
```

```
  FLGELF = 0
  IF(CMT.EQ.3) FLGELF = 1

  IPRED=F
  IRES=DV-IPRED
  TMPRES1 = EPS(1)*(1-FLGELF)
  TMPRES2 = EPS(2)*FLGELF
  Y = F + F*TMPRES1 + TMPRES2
  IWRES=IRES/IPRED

$THETA
  (0,3.00)             ; CL      ; L/hr
  (0,10.5)             ; VC      ; L
  (0,0.550)            ; CLD     ; L/hr
  (0,3.0)              ; K13     ; 1/hr
  (0,6.0)              ; K30     ; 1/hr
  (0.3)                ; CL_CLCR ;

$OMEGA BLOCK(1) 0.1    ; IIV_CL
$OMEGA BLOCK(1) SAME   ; IIV_VC
$OMEGA BLOCK(1) SAME   ; IIV_K30

$SIGMA
0.015                  ; Prop_RV_PLASMA
0.200                  ; Add_RV_ELF

$EST METHOD=CONDITIONAL INTERACTION MAXEVAL=9999 PRINT=5 NOABORT SIGL=6 NSIG=2
     NOTHETABOUNDTEST NOOMEGABOUNDTEST NOSIGMABOUNDTEST

$COV UNCONDITIONAL

$TABLE ID TIME TSLD CMT AMT IPRED IWRES IRES CWRES CP CPERIPH CELF WTKG AGE SEXF BSA
       CLCR CL VC K30 NOPRINT ONEHEADER FILE=popPK-tobra.tbl
```

NONMEM dataset used to develop the population PK model for tobramycin

ID	TIME	TSLD	AMT	RATE	ADDL	II	DV	CMT	EVID	MDV	WTKG	AGE	SEXF	HTCM	SCR
1	0		73	146	42	8		1	1	1	73	72	0	164	0.9
1	336.5	0.5					6.08	1	0	0	73	72	0	164	0.9
1	338	2					4.57	1	0	0	73	72	0	164	0.9
1	340	4					2.87	1	0	0	73	72	0	164	0.9
1	340	4					3.4	3	0	0	73	72	0	164	0.9
1	344	8					1.47	1	0	0	73	72	0	164	0.9
2	0		68	136	42	8		1	1	1	68	49	0	148	1
2	336.5	0.5					8.37	1	0	0	68	49	0	148	1
2	336.5	0.5					2.54	3	0	0	68	49	0	148	1
2	338	2					6	1	0	0	68	49	0	148	1
2	340	4					3.29	1	0	0	68	49	0	148	1
2	344	8					1.16	1	0	0	68	49	0	148	1
3	0		68	136	42	8		1	1	1	68	36	0	190	1
3	336.5	0.5					9.09	1	0	0	68	36	0	190	1
3	336.5	0.5					2.57	3	0	0	68	36	0	190	1
3	338	2					4.72	1	0	0	68	36	0	190	1
3	340	4					2.67	1	0	0	68	36	0	190	1
3	344	8					1.01	1	0	0	68	36	0	190	1
4	0		50	100	42	8		1	1	1	50	34	1	157	1
4	336.5	0.5					6.42	1	0	0	50	34	1	157	1
4	338	2					4.37	1	0	0	50	34	1	157	1
4	340	4					2.54	1	0	0	50	34	1	157	1
4	340	4					0.93	3	0	0	50	34	1	157	1
4	344	8					0.87	1	0	0	50	34	1	157	1
5	0		71	142	42	8		1	1	1	71	28	1	159	0.9
5	336.5	0.5					4.58	1	0	0	71	28	1	159	0.9
5	338	2					2.57	1	0	0	71	28	1	159	0.9
5	340	4					1.13	1	0	0	71	28	1	159	0.9
5	344	8					0.27	1	0	0	71	28	1	159	0.9
5	344	8					0.43	3	0	0	71	28	1	159	0.9
6	0		67	134	42	8		1	1	1	67	44	1	159	0.6
6	336.5	0.5					1.46	3	0	0	67	44	1	159	0.6
6	338	2					2.54	1	0	0	67	44	1	159	0.6
6	340	4					1.15	1	0	0	67	44	1	159	0.6
6	344	8					0.31	1	0	0	67	44	1	159	0.6
7	0		65	130	42	8		1	1	1	65	48	0	158	1.1
7	336.5	0.5					6.18	1	0	0	65	48	0	158	1.1
7	338	2					4.36	1	0	0	65	48	0	158	1.1
7	338	2					2.25	3	0	0	65	48	0	158	1.1

ID	TIME	TSLD	AMT	RATE	ADDL	II	DV	CMT	EVID	MDV	WTKG	AGE	SEXF	HTCM	SCR
7	340	4					1.69	1	0	0	65	48	0	158	1.1
7	344	8					0.54	1	0	0	65	48	0	158	1.1
8	0		84	168	42	8		1	1	1	84	56	1	147	0.6
8	336.5	0.5					7.09	1	0	0	84	56	1	147	0.6
8	336.5	0.5					2.34	3	0	0	84	56	1	147	0.6
8	338	2					4.83	1	0	0	84	56	1	147	0.6
8	340	4					2.32	1	0	0	84	56	1	147	0.6
8	344	8					0.61	1	0	0	84	56	1	147	0.6
9	0		77	154	42	8		1	1	1	77	25	0	176	1.2
9	336.5	0.5					7.74	1	0	0	77	25	0	176	1.2
9	338	2					5.49	1	0	0	77	25	0	176	1.2
9	338	2					1.56	3	0	0	77	25	0	176	1.2
9	340	4					4.17	1	0	0	77	25	0	176	1.2
9	344	8					0.86	1	0	0	77	25	0	176	1.2
10	0		60	120	42	8		1	1	1	60	45	1	159	0.7
10	336.5	0.5					8.47	1	0	0	60	45	1	159	0.7
10	338	2					6.64	1	0	0	60	45	1	159	0.7
10	340	4					2.57	1	0	0	60	45	1	159	0.7
10	340	4					1.17	3	0	0	60	45	1	159	0.7
10	344	8					1.56	1	0	0	60	45	1	159	0.7
11	0		62	124	42	8		1	1	1	62	39	0	156	1
11	336.5	0.5					8.5	1	0	0	62	39	0	156	1
11	338	2					3.98	1	0	0	62	39	0	156	1
11	340	4					1.76	1	0	0	62	39	0	156	1
11	344	8					0.6	1	0	0	62	39	0	156	1
11	344	8					0.45	3	0	0	62	39	0	156	1
12	0		68	136	42	8		1	1	1	68	47	0	168	1
12	336.5	0.5					8.07	1	0	0	68	47	0	168	1
12	338	2					3.61	1	0	0	68	47	0	168	1
12	340	4					1.82	1	0	0	68	47	0	168	1
12	340	4					1	3	0	0	68	47	0	168	1
12	344	8					0.48	1	0	0	68	47	0	168	1
13	0		59	118	42	8		1	1	1	59	23	0	172	0.9
13	336.5	0.5					4.92	1	0	0	59	23	0	172	0.9
13	338	2					2.14	1	0	0	59	23	0	172	0.9
13	340	4					1.08	1	0	0	59	23	0	172	0.9
13	344	8					0.93	1	0	0	59	23	0	172	0.9
13	344	8					1.15	3	0	0	59	23	0	172	0.9
14	0		70	140	42	8		1	1	1	70	67	0	168	0.7
14	336.5	0.5					6.63	1	0	0	70	67	0	168	0.7
14	338	2					3.56	1	0	0	70	67	0	168	0.7
14	338	2					2.12	3	0	0	70	67	0	168	0.7

ID	TIME	TSLD	AMT	RATE	ADDL	II	DV	CMT	EVID	MDV	WTKG	AGE	SEXF	HTCM	SCR
14	340	4					2.02	1	0	0	70	67	0	168	0.7
14	344	8					0.72	1	0	0	70	67	0	168	0.7
15	0		67	134	42	8		1	1	1	67	41	0	163	0.9
15	336.5	0.5					6.39	1	0	0	67	41	0	163	0.9
15	338	2					2.71	1	0	0	67	41	0	163	0.9
15	338	2					0.76	3	0	0	67	41	0	163	0.9
15	340	4					1.52	1	0	0	67	41	0	163	0.9
15	344	8					0.76	1	0	0	67	41	0	163	0.9
16	0		69	138	42	8		1	1	1	69	42	0	169	1.1
16	336.5	0.5					4.96	1	0	0	69	42	0	169	1.1
16	338	2					3.19	1	0	0	69	42	0	169	1.1
16	340	4					1.66	1	0	0	69	42	0	169	1.1
16	344	8					0.41	1	0	0	69	42	0	169	1.1
16	344	8					1.04	3	0	0	69	42	0	169	1.1
